# Ovarian cancer prevention by opportunistic salpingectomy is a new de facto standard in Germany

**DOI:** 10.1007/s00432-023-04578-5

**Published:** 2023-02-27

**Authors:** I. B. Runnebaum, A. Kather, J. Vorwergk, J. J. Cruz, A. R. Mothes, C. R. Beteta, J. Boer, M. Keller, M. Pölcher, A. Mustea, J. Sehouli

**Affiliations:** 1 Department of Gynecology and Reproductive Medicine, Jena University Hospital, Friedrich Schiller-University Jena, Am Klinikum 1, 07747 Jena, Germany; 2 Department of Gynecology with Center for Oncological Surgery, Charité-University Medicine Berlin, Campus Virchow-Klinikum, Augustenburger Platz 1, 13353 Berlin, Germany; 3 Nord-Ostdeutsche Gesellschaft für Gynaekologische Onkologie (NOGGO e.V.), Schwedenstraße 9, 13359 Berlin, Germany; 4 Department of Gynecologic Oncology and Minimal Invasive Surgery, Rotkreuzklinikum München Frauenklinik, Taxisstraße 3, 80637 München, Germany; 5 Gynecology and Gynecologic Oncology, University Hospital Bonn, Venusberg-Campus 1, 53127 Bonn, Germany; 6 Department of Obstetrics and Perinatal Medicine, Bonn University Hospital, Sigmund Freud Street 25, 53127 Bonn, Germany; 7 Department of Gynecology, St. Georg Hospital Eisenach, Academic Teaching Hospital of University of Jena, Muehlhaeuser Str. 94, 99817 Eisenach, Germany

**Keywords:** Opinion, Case number analysis, Fallopian tubes, Opportunistic salpingectomy, Tubal sterilization, Definitive contraception, Hysterectomy, Cancer prevention

## Abstract

**Purpose:**

The most prevalent and aggressive subtype of epithelial ovarian carcinoma (EOC), high-grade serous carcinoma (HGSC), originates in many cases from the fallopian tubes. Because of poor prognosis and lack of effective screening for early detection, opportunistic salpingectomy (OS) for prevention of EOC is being implemented into clinical routine in several countries worldwide. Taking the opportunity of a gynecological surgery in women at average cancer risk, extramural fallopian tubes are completely resected preserving the ovaries with their infundibulopelvic blood supply. Until recently, only 13 of the 130 national partner societies of the International Federation of Obstetrics and Gynecology (FIGO) have published a statement on OS. This study aimed to analyze the acceptance of OS in Germany.

**Methods:**

(1) Survey of German gynecologists in 2015 and 2022 by the Department of Gynecology of the Jena University Hospital in co-operation with the Department of Gynecology at Charité-University Medicine Berlin with support of NOGGO e. V. and AGO e. V. (2) Salpingectomy numbers in Germany for years 2005–2020 as retrieved from the Federal Statistical Office of Germany (Destatis).

**Results:**

(1) Survey: Number of participants was 203 in 2015 and 166 in 2022, respectively. Nearly all respondents (2015: 92%, 2022: 98%) have already performed bilateral salpingectomy without oophorectomy in combination with benign hysterectomy with the intention to reduce the risk for malignant (2015: 96%, 2022: 97%) and benign (2015: 47%, 2022: 38%) disorders. Compared to 2015 (56.6%), considerably more survey participants performed OS in > 50% or in all cases in 2022 (89.0%). Recommendation of OS for all women with completed family planning at benign pelvic surgery was approved by 68% in 2015 and 74% in 2022. (2) Case number analysis: In 2020, four times more cases of salpingectomy were reported by German public hospitals compared to 2005 (*n* = 50,398 vs. *n* = 12,286). Of all inpatient hysterectomies in German hospitals in 2020, 45% were combined with salpingectomy, and more than 65% in women at the age of 35 to 49 years.

**Conclusion:**

Mounting scientific plausibility regarding involvement of fallopian tubes in the pathogenesis of EOC led to change of clinical acceptance of OS in many countries including in Germany. Case number data and widespread expert judgment demonstrate that OS has become a routine procedure in Germany and a de facto standard for primary prevention of EOC.

**Supplementary Information:**

The online version contains supplementary material available at 10.1007/s00432-023-04578-5.

## Introduction

In Germany, more than 7000 women are newly diagnosed with ovarian cancer annually and more than 5000 succumb from this disease (German Centre for Cancer Registry Data 2022). This happens despite extensive treatment including cytoreductive surgery with the attempt to remove all macroscopic tumor followed by chemotherapies and pricey maintenance therapy. Effective early detection is not available for ovarian cancer. During the first decade of this century, scientific evidence was given for an extra-ovarian origin of high-grade serous ovarian carcinoma (HGSOC) (Kurman and Shih 2010). HGSOC is usually diagnosed at late stages of disease, has very unfavorable prognosis and accounts for more than 50% of ovarian carcinoma cases (Buttmann-Schweiger and Kraywinkel 2019).

In prophylactically removed fallopian tubes of women with hereditary breast and ovarian carcinoma (carriers of BRCA mutation), but also in fallopian tubes of women with sporadic ovarian cancer, serous tubal intraepithelial carcinoma (STIC) was noticed in a substantial number of cases already comprising driver mutations as in HGSOC. STIC was then recognized as a precursor lesion of HGSOC (Piek et al. 2001; Medeiros et al. 2006; Kindelberger et al. 2007; Carlson et al. 2008; Jarboe et al. 2009; Kurman and Shih 2010; Kurman et al. 2011). This led to the new classification “Tubo-ovarian” cancer in the WHO guidelines 2020 (McCluggage et al. 2022). Salpingectomy usually refers to removing the extramural part of the fallopian tubes completely including isthmus, ampulla and fimbriae bilaterally while preserving the ovaries with their infundibulopelvic blood supply. Several retrospective population-based studies proved salpingectomy to be a protective factor for woman at average risk for ovarian cancer (Lessard-Anderson et al. 2014; Falconer et al. 2015, 2021; Madsen et al. 2015; van Lieshout, L A M et al. 2021). Recent cost-effectiveness analyses in the USA estimated that opportunistic salpingectomy (OS) in combination with benign hysterectomy or as a permanent contraception method could prevent at least 14.5% of ovarian cancer related deaths and could reduce up to $445 million healthcare costs annually (Dilley et al. 2017; Subramaniam et al. 2019; Naumann et al. 2021).

Just 13 national societies of the 130 member societies of the International Federation of Obstetrics and Gynecology (FIGO) have published statements on OS (Ntoumanoglou-Schuiki et al. 2018). Of these, nine statements generally recommend to offer OS to all women, who have completed their family planning and undergo pelvic surgery. Four statements, including the one published by the DGGG, the Germany Society for Gynecology and Obstetrics (Pölcher et al. 2015; German Guideline Program in Oncology 2022), have been reluctant to generally recommend OS. This is justified by the missing direct evidence for the protective effect of OS. Yet, appropriately controlled prospective studies are difficult to perform due to the low prevalence of OC (life time risk 1 out of 70) and the lengthy time period between typical age of intervention for pelvic diseases and the disease onset mostly in the elderly. However, the time point of hysterectomy appears to be ideal for OS considering that the prevalence of hysterectomy in women at the age of 60 years and above is more than 30% (Prütz et al. 2013) while 70% of all ovarian carcinoma cases are observed in this age group (Buttmann-Schweiger and Kraywinkel 2019).

At the Jena University Hospital, we started to perform OS routinely in fall of 2005. With the hesitancy of institutions and commissions to realize the prophylactic aspects of OS in the past two decades, we now analyze the evolving real-world situation in Germany: whether OS is being implemented in daily clinical practice, still without published guideline recommendation and alongside ambivalent expert statements. For this purpose, we conducted (1) a survey of German gynecologists in years 2015 and 2022 and (2) an analysis of salpingectomy case numbers during 2005 and 2020 in German hospitals.

## Methods

This project is an initiative of the Department of Gynecology at Jena University Hospital together with the Department of Gynecology at Charité-University Medicine Berlin in cooperation with the Northeastern German Society of Gynecologic Oncology („Nord-Ostdeutsche Gesellschaft für Gynaekologische Onkologie”, NOGGO e. V.) and is supported by the ovary commission of the AGO e. V. (“Arbeitsgemeinschaft Gynaekologische Onkologie”) as part of the German Society for Gynecology and Obstetrics (“Deutsche Gesellschaft für Gynaekologie und Geburtshilfe e. V.”, DGGG).

The questionnaire (German version provided in supplemental material 1) was developed based on a survey conducted in 2013 by Gill and Mills in the USA (Gill and Mills 2013). Using a total of 25 questions, setting and size of hospital, as well as baseline data (position, sex, clinical experience) and opinion regarding opportunistic salpingectomy of respondents were collected. Selection of multiple answers and skipping of questions was allowed. After obtaining approval from the local ethics committee, the anonymous survey was conducted using „SurveyMonkey “ (Palo Alto, CA) in 2015 and in 2022. In 2015, invitation and questionnaire were distributed via email to all NOGGO e. V. registered German hospitals providing gynecological pelvic surgery (635 hospitals). The survey was open from July 3rd to September 30th 2015. Due to preferences by NOGGO e. V. members in the year 2022, direct survey invitation via email was no longer used, the invitation and link to the survey was placed on the homepage and distributed via the newsletter of NOGGO e. V. instead. The survey was open from August 8th to October 16th 2022. To increase awareness of the survey, the link was additionally distributed via email to gynecologic oncologists of the Ovarian and Uterine Commissions of the AGO e. V., the Study Group for Urogynecology and Plastic Pelvic Floor Reconstruction (“Arbeitsgemeinschaft für Urogynaekologie und plastische Beckenbodenrekonstruktion”, AGUB e.V.), “Arbeitsgemeinschaft Gynaekologische und Geburtshilfliche Endoskopie e.V. (AGE e. V.)” and to the clinical management offices of hospitals in the region Berlin and Brandenburg. The 2022 survey was advertised in scientific presentations at the meeting of “German speaking university lecturers” held in Linz, Austria, September 17th, 2022 and at the 64th Congress of the German Society for Gynecology and Obstetrics (Deutsche Gesellschaft für Gynaekologie und Geburtshilfe e. V., DGGG) held in Munich, Germany, October 12th–15th, 2022. The anonymously answered questionnaires were collected in SurveyMonkey followed by descriptive analysis of number (n) and proportion (%) of respondents for each item.

Numbers of salpingectomies (OPS 5–661.*), salpingo-oophorectomies (OPS 5–653.*), tubal ligations (OPS 5–663.*) and benign hysterectomies (OPS 5–682.* + OPS 5–683.*) which were conducted as inpatient cases (including deceased and short-stay cases) in public hospitals in Germany as single or combined procedures from 2005 to 2020 were retrieved from the Federal Statistical Office of Germany (“Statistisches Bundesamt”, gesundheit@destatis.de, special analysis of DRG cases). Duplicates on the level of the four-digit OPS code were excluded.

## Results

### Survey

A Germany-wide survey was conducted in 2015 (203 respondents) and again in 2022 (166 respondents) to analyze the development of approaches of experienced gynecologists toward opportunistic salpingectomy.

Baseline data of hospitals and respondents are shown in Table 1. The majority of respondents work in the fields of general/benign gynecology (2015: 82.7%, 2022: 77.7%) and/or gynecological oncology (2015: 84.7%, 2022: 63.3%) and have spent 10 or more years in medical services (2015: 96.5%, 2022: 87.3%). The survey was predominantly answered by chief or senior specialists (2015: 99.5%, 2022: 89.8%). While 2015 only 22.3% of respondents were female, this proportion increased to 47.0% in the year 2022. Compared to 2015, a higher proportion of respondents worked at an academic institution (4.0 vs 24.9%) and/or at a certified gynecologic cancer center (26.7 vs 52.7%) in 2022.

**Table 1:** Survey opportunistic salpingectomy—Demographic data of survey participants. Multiple answers were allowed for all questions

Questionnaire item	2015 (*n* = 203)		2022 (*n* = 166)	
	*n*	%	*n*	%
Area of specialty				
General/Benign Gynecology	167	82.7	129	77.7
Gynecological Oncology	171	84.7	105	63.3
Urogynecology	98	48.5	69	41.6
Reproductive Medicine	5	2.5	1	0.6
Obstetrics	109	54	43	25.9
Skipped question	1		0	
Who is answering this questionnaire?				
Chief Physician/Head of Department	161	79.7	71	42.8
Senior Physician/Assistant Medical Director	40	19.8	78	47
Attending Physician	0	0	4	2.4
Medical specialist	2	1	10	6.0
Assistant Physician with professional experience	1	0.5	6	3.6
Skipped question	1		0	
Years in practice				
≥ 20	141	69.8	90	54.2
10–19	54	26.7	55	33.1
5–9	7	3.5	19	11.5
< 5	0	0	3	1.8
Skipped question	1		0	
Sex				
Male	157	77.7	88	53
Female	45	22.3	78	47
Skipped question	1		0	
Service level of hospital				
Tertiary care hospital	37	18.6	59	36.2
Secondary care hospital	66	33.2	51	31.3
Primary care hospital	97	48.7	48	29.5
Day surgery unit	0	0	8	4.9
Skipped question	4		3	
Practice setting				
Academic institution	8	4	41	24.9
Public hospital	97	48.5	59	35.8
Non-profit private hospital (ecclesiastical provider)	56	28	43	26.1
Private hospital	41	20.5	20	12.1
Private practice clinic	0	0	7	4.2
Skipped question	3		1	
Certified gynecological cancer centre (Except Breast Cancer Centre)				
Yes	54	26.7	87	52.7
No	148	73.3	78	47.3
Skipped question	1		1	

**Fig. 1:**
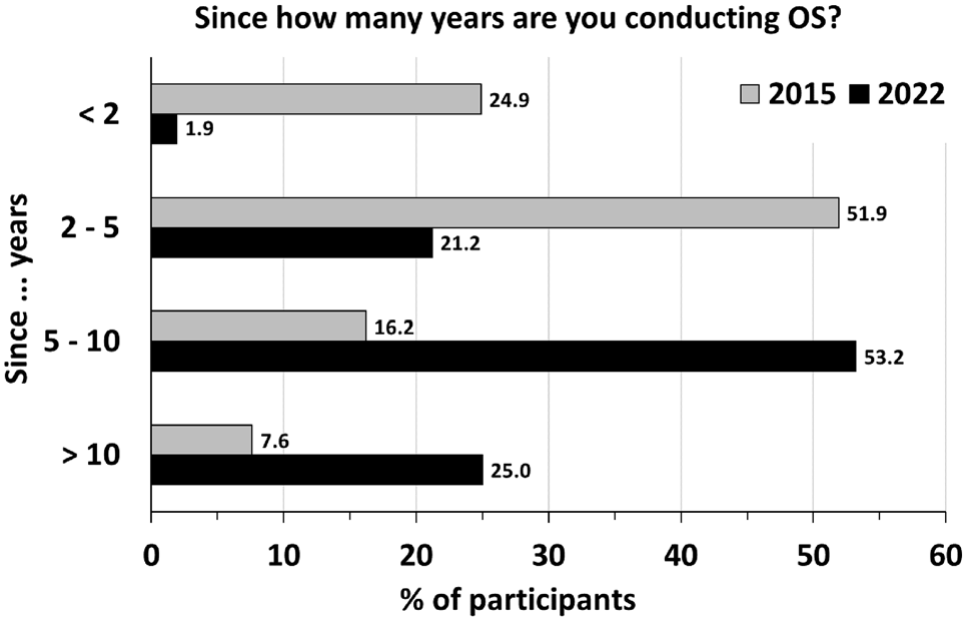
Proportion of respondents of the survey conducted in years 2015 and 2022, who perform OS since < 2 years, 2–5 years, 5–10 years and > 10 years

Nearly all respondents (2015: 92.0%, 2022: 98.1%) had already performed bilateral salpingectomy without oophorectomy in association with a hysterectomy for benign indications and no pathological findings at fallopian tubes (Table 2). The key intention for OS was cancer risk reduction in general (2015: 96.2%, 2022: 97.4%, Table 2) as well as in patients with increased risk for breast and ovarian cancer (2015: 40.0%, 2022: 45.8%), followed by the intention to prevent hydrosalpinx (2015: 96.2%, 2022: 97.4%). Compared to 2015 (56.6%), considerably more German gynecologists performed OS in > 50% of or in all cases in 2022 (89.0%, Table 2). While in 2015 the majority of respondents indicated that they performed OS in the past 2–5 years (corresponding to years 2010–2013), this shifted to 5–10 years in 2022 (corresponding to years 2012–2017, Table 2 and Fig. 1). Few respondents have never performed OS (2015: 8.0%, 2022: 1.9%) arguing that it might increase the risk of surgical complications (2015: 52.0%, 2022: 37.5%), might prolong the operation (2015: 24.0%, 2022: 25.0%) or that it adds no benefit (2015: 36.0%, 2022: 37.5%, Table 2).

**Table 2:** Survey opportunistic salpingectomy – Opinion regarding OS in conjunction with hysterectomy. Multiple answers were allowed for all questions

Questionnaire item	2015 (*n* = 203)		2022 (*n* = 166)	
	*n*	%	*n*	%
Did you ever perform bilateral salpingectomy in association with a hysterectomy without oophorectomy for benign indications and no pathological findings at fallopian tubes?				
Yes	185	92.0	155	98.1
No	16	8.0	3	1.9
Skipped question	2		8	
If yes, since:				
> 10 years	14	7.6	39	25
5–10 years	30	16.2	83	53.2
2–5 years	96	51.9	33	21.2
< 2 years	46	24.9	3	1.9
Skipped question	18		10	
If yes, how frequently?				
< 10% of cases	31	17	5	3.3
10–50% of cases	48	26.4	16	10.4
> 50% of cases	62	34.1	77	50
At all times	41	22.5	60	39
Skipped question	21		12	
If yes, why did you combine hysterectomy for benign indications with bilateral salpingectomy?				
To decrease the risk of pelvic pain	16	8.6	14	9.0
To decrease the risk of cancer	178	96.2	151	97.4
To decrease the risk of cancer in a patient with increased risk for breast and ovarian cancer	74	40.0	71	45.8
To decrease the risk of reoperation	33	17.8	15	9.7
To decrease the risk of formation of hydrosalpinges	86	46.5	59	38.0
Skipped question	18		11	
If no, why not?				
It increases the risk of operative complications	13	52	3	37.5
It increases operative time	6	24	2	25
Bilateral salpingectomy does not decrease the risk of cancer	2	8.0	2	25.0
The risk of reoperation is the same regardless of whether bilateral salpingectomy is performed	3	12.0	1	12.5
There is no benefit	9	36.0	3	37.5
Skipped question	178		158	
Benefit and risk: With respect to the risks and benefits of bilateral salpingectomy in association with a hysterectomy without oophorectomy for benign indications, I believe				
The benefits are worth the risk	184	93.4	151	96.8
There is no benefit	13	6.6	5	3.2
Skipped question	6		10

Predominant anamnestic factors, which impacted the decision to indicate bilateral salpingectomy, were increased risk for gynecologic cancer (2015 and 2022: 83.3), confirmed gBRCA mutation (2015: 64.9%, 2022: 74.2%) and patient age (2015: 63.1%, 2022: 79.6%, Table S1). Most respondents would perform OS starting from a patient age of 40 years (2015: 49.4%, 2022: 51.8%) and others starting from an age of 30 years (2015: 32.6%, 2022: 35.0%, Table S1).

Weighing the risks and benefits of OS, the vast majority of respondents believe that the benefits outweigh the potential risks (2015: 93.4%, 2022: 96.8%, Table 2). The most important benefit is seen in the decrease of the risk of fallopian tube, ovarian or primary peritoneal cancer in the future (2015: 89.0%, 2022: 93.7%), followed by the risk reduction regarding tubal disease (2015: 42.5%, 2022: 35.4%, Table 3). Most gynecologists do not believe that there are additional risks performing OS in addition to hysterectomy or as an alternative to tubal ligation for sterilization (2015: 74.9, 2022: 79.0%, Table 3). The percentage of respondents thinking that bilateral salpingectomy should be recommended to women who have completed family planning and are undergoing any endoscopic, abdominal or vaginal surgery with accessible fallopian tubes increased from 68.2% in 2015 to 74.0% in 2022 (Table 3).

**Table 3:** Survey opportunistic salpingectomy – Opinion regarding risks and benefit, recommendation. Multiple answers were allowed for all questions

Questionnaire item	2015 (*n* = 203)		2022 (*n* = 166)	
	*n*	%	*n*	%
Additional risks: Do you believe there are additional risks to performing bilateral salpingectomy in addition to hysterectomy or other form of tubal sterilization?				
Yes	50	25.1	33	21.0
No	149	74.9	124	79.0
Skipped question	4		9	
Most important benefit: What do you believe is the most important benefit of elective bilateral salpingectomy?				
It decreases the risk of fallopian tube, ovarian or primary peritoneal cancer in the future	178	89.0	148	93.7
It decreases the risk of pelvic pain in the future	14	7.0	13	8.2
It decreases the risk of tubal disease in the future	85	42.5	56	35.4
I do not believe that it is beneficial	9	4.5	1	0.6
Skipped question	3		8	
Do you think, bilateral salpingectomy should be recommended to each woman, who has completed childbearing and is undergoing endoscopic, abdominal or vaginal surgery with accessible fallopian tubes?				
Yes	135	68.2	114	74.0
No	63	31.8	40	26.0
Skipped question	5		12	

Most respondents believe that bilateral salpingectomy is the most effective method for permanent sterilization (2015: 65.3%, 2022: 79.6%, Table 4). While in 2015 only 26.4% of survey participants stated bilateral salpingectomy as their preferred sterilization method, this proportion increased to 56.8% in 2022. Accordingly, the percentage of respondents, which prefer bipolar coagulation of tubes decreased from 87.3 to 55.5%. Reasons for not considering bilateral salpingectomy were specified as increased risk of intraoperative complications (2015: 48.2%, 2022: 57.9%), increased intraoperative time (2015: 30.6%, 2022: 23.7%) and as being not superior to other methods for sterilization (2015: 54.1%, 2022: 39.5%, Table 4). Gynecological surgeons consider bilateral salpingectomy as a sterilization procedure to decrease the risk of cancer in general (2015: 66.7%, 2022: 81.2%) and in patients with increased risk for breast and ovarian cancer (2015: 68.7%, 2022: 73.4%), as well as in case of fallopian tube disease (2015: 78.8%, 2022: 75.3%) and previous failed sterilization (2015: 55.6%, 2022: 60.4%, Table 4).

**Table 4:** Survey opportunistic salpingectomy – Opinion regarding OS for permanent sterilization. Multiple answers were allowed for all questions

Questionnaire item	2015 (*n * = 203)		2022 (*n *= 166)	
	*n*	%	*n*	%
What do you believe is the most effective method for tubal sterilization for a woman older than 35 years?				
Bipolar coagulation with or without transection of tubes	72	36.7	42	26.8
Bilateral salpingectomy	128	65.3	125	79.6
Filshie Clips (Titanium clip with silicone rubber surface coating)	0	0	0	0
Silicone rubber band application (Falope ring)	0	0	0	0
No difference in any of the above methods	26	13.3	11	7.0
Skipped question	7		9	
For which indications would you consider performing a total bilateral salpingectomy as a sterilization procedure?				
Fallopian tube disease	156	78.8	116	75.3
To decrease the risk of cancer	132	66.7	125	81.2
In case of increased risk for breast and ovarian cancer, in order to decrease cancer risk	136	68.7	113	73.4
To decrease the risk of pelvic pain	14	7.1	22	14.3
To decrease failure of sterilization in a patient in whom a sterilization procedure has already failed	110	55.6	93	60.4
I would not consider performing bilateral salpingectomy as a sterilization procedure	13	6.6	6	3.9
Skipped question	5		12	
Why would you not consider performing a bilateral salpingectomy as a sterilization procedure?				
Increased intraoperative time	26	30.6	9	23.7
Increased risk of intraoperative complications	41	48.2	22	57.9
It is not superior to other methods of sterilization	46	54.1	15	39.5
It is not beneficial	2	2.4	3	7.9
Skipped question	118		128	
What is your preferred method of tubal sterilization?				
Bipolar coagulation with or without transection of tubes	172	87.3	86	55.5
Bilateral salpingectomy	52	26.4	88	56.8
Filshie Clips (Titanium clip with silicone rubber surface coating)	0	0	1	0.7
Silicone rubber band application (Falope ring)	0	0	0	0
Skipped question	6		11	

In 2022, considerably more survey participants state that histopathological examination of removed tubes is carried out according to the special SEE-FIM protocol for the detection of STIC (2015: 27.5%, 2022: 68.0%, Table S2).

### Development of salpingectomy numbers in Germany 2005–2020

Salpingectomy procedures which were conducted in German public hospitals increased almost fourfold from 2005 (12,286) to 2020 (50,398). Figures were stable until 2011 (12,167) when rates exponentially increased to at least 5000 cases per year until 2018 (54,382). In 2019, 55,293 cases were reported with a slight decrease in 2020. Ranking the most frequent surgeries in women in Germany in 2020, salpingectomy was found on position #40. Consequently, salpingectomies were performed more frequently as compared to appendectomies in the female population (#41; 49,729 cases) according to Federal Health Reporting (Federal Statistical Office of Germany).

The number of tubal sterilizations, which were carried out as tubal ligation, moderately increased between 2005 (6,943 cases) and 2020 (9,243 cases). Intriguingly, compared to 2005 (146,665 cases) the number of inpatient hysterectomies for benign indications nearly halved (2020: 83,174 cases).

**Fig. 2:**
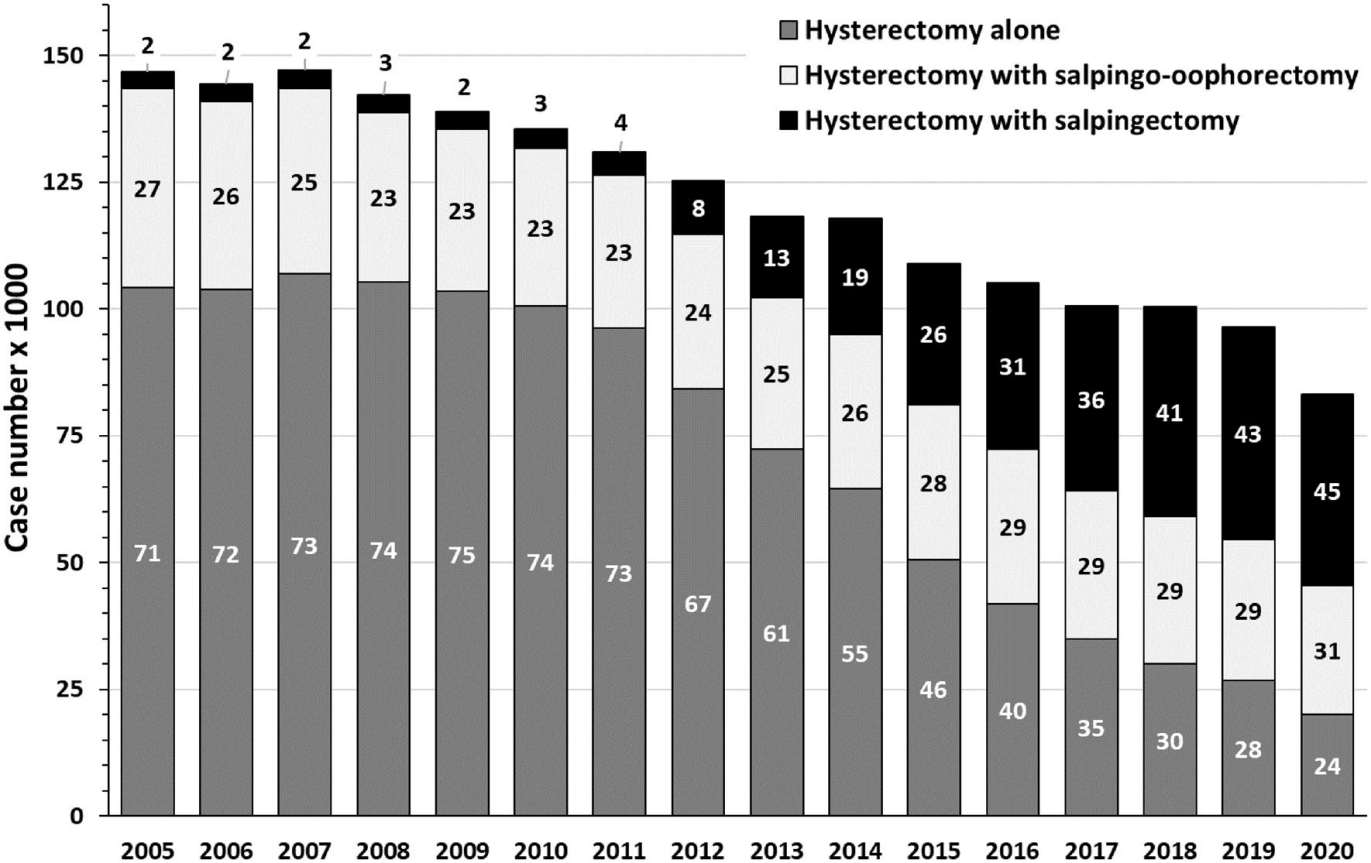
Hysterectomies 2005–2020, performed alone or combined with salpingectomy or salpingo-oophorectomy. Numbers within columns indicate percentages. Data according to Federal Statistical Office of Germany ("Statistisches Bundesamt", gesundheit@destatis.de, special analysis of DRG cases), own presentation

The proportion of benign hysterectomies combined with salpingectomy was as low as 4% until the year 2011 (4,593 combined procedures out of 130,965 hysterectomies, Fig. 2). Thereafter, the proportion increased continuously to 45% in 2020 (procedures including OS: 37,732; Fig. 2). In 23–31% of cases, hysterectomies were performed along with complete bilateral salpingo-oophorectomy, with only moderate decline over the period studied (2005: 39,289, 2011: 30,223, 2020: 25,425, Fig. 2).

**Fig. 3:**
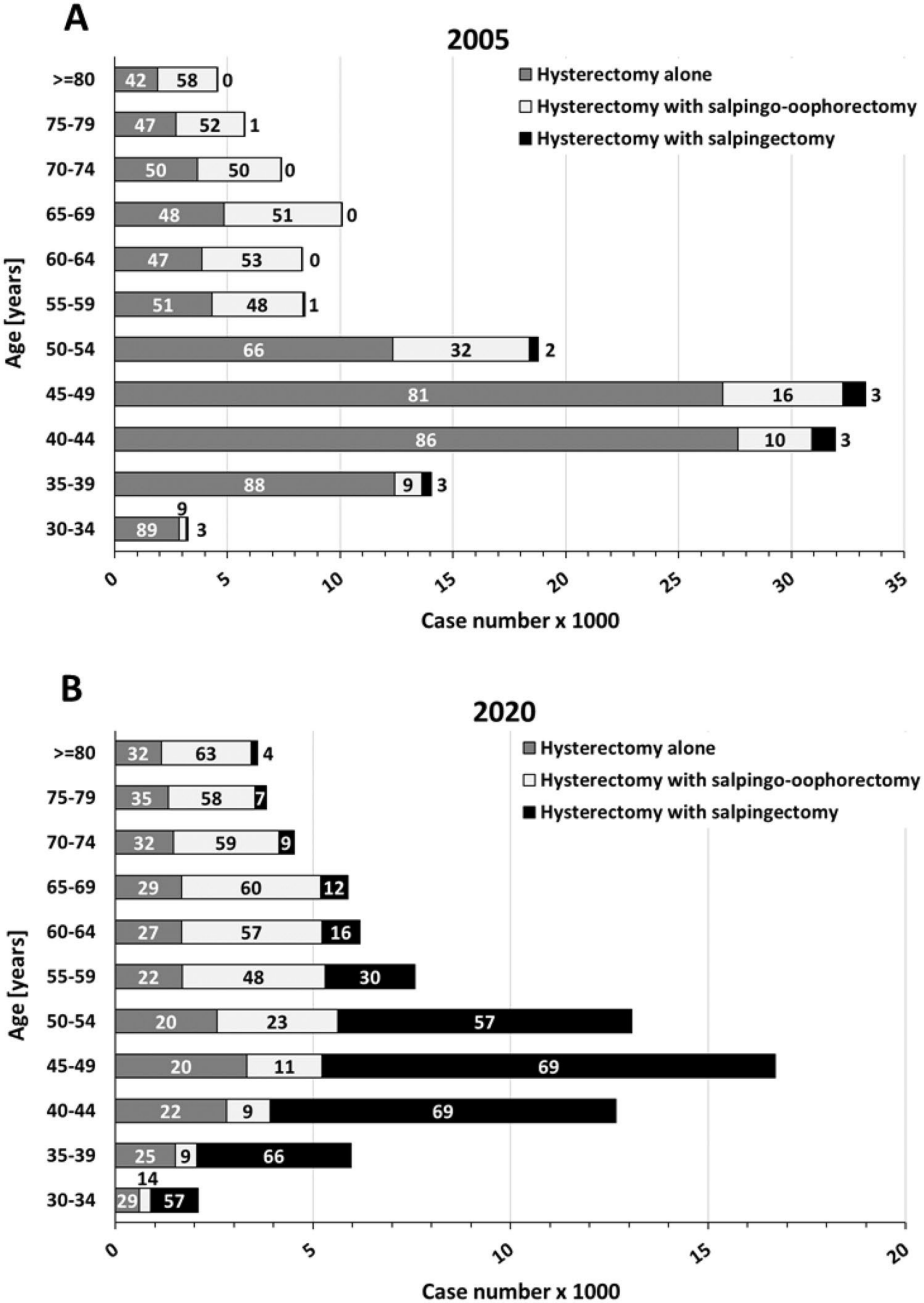
Hysterectomies in years 2005 (**A**) and 2020 (**B**), which were performed alone or in combination with salpingectomy or salpingo-oophorectomy, depicted for different age classes. Numbers within columns indicate percentages. Data according to Federal Statistical Office of Germany („Statistisches Bundesamt “, gesundheit@destatis.de, special analysis of DRG cases), own presentation

The increase of combined procedures occurred predominantly in premenopausal patients, as shown in an analysis of age-dependent case numbers in 2020 compared to 2005 (Fig. 3). In contrast to the beginning of the century, today hysterectomy combined with salpingectomy is the preferred approach (57–69%) in women 30–54 years of age. In patients older than 60 years, complete adnexectomy (bilateral salpingo-oophorectomy) is performed in the majority of cases, without much change in the studied time period.

**Fig. 4:**
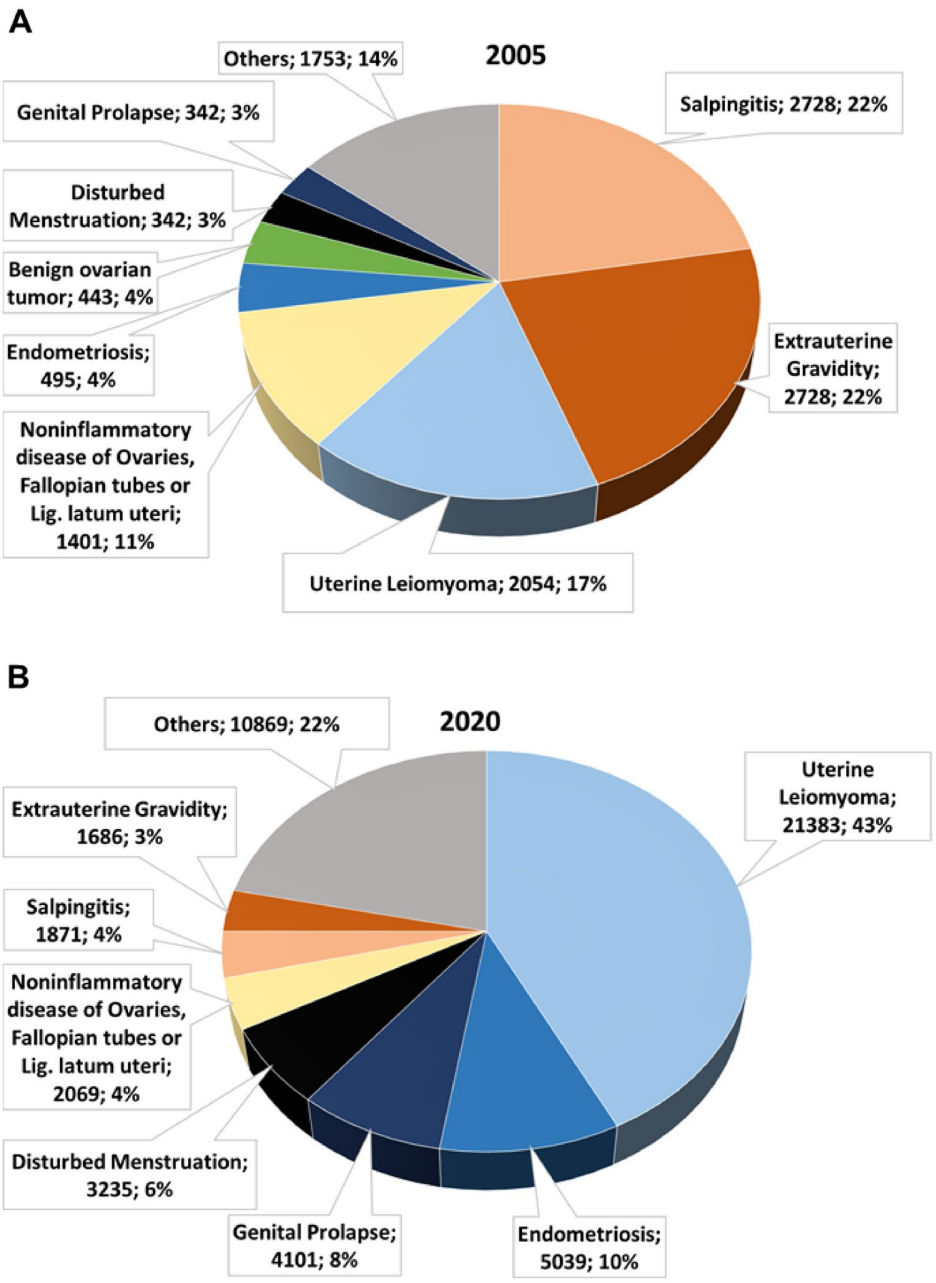
Main diagnosis, which was registered together with salpingectomies in 2005 (**A**) and 2020 (**B**) presented with absolute case numbers and percentages. Data according to Federal Statistical Office of Germany ("Statistisches Bundesamt", gesundheit@destatis.de, special analysis of DRG cases), as analyzed and illustrated by our study

Furthermore, we searched for main indications, which were registered together with salpingectomies in 2005 and 2020 (Fig. 4). In 2020, as many as two thirds of salpingectomies were performed as OS at the occasion of benign indications for hysterectomy due to uterine leiomyoma, endometriosis, genital prolapse or abnormal menstrual bleeding, but only 11% were indicated because of fallopian tube pathologies such as noninflammatory disease of ovaries or of fallopian tubes or of the uterine broad ligament, salpingitis or ectopic pregnancy. Back in 2005, the picture was completely different: leading indications were salpingitis and extrauterine gravidity (each 22%).

## Discussion

The vast majority of our survey participants perform opportunistic salpingectomy combined with benign hysterectomy in most cases. They judge that complete removal of fallopian tubes is beneficial regarding primary prevention of high-grade serous tubo-ovarian cancer (HGSTOC) and benign tubal pathologies. The repeated survey in 2022 reveals this attitude being further established since 2015. Support for salpingectomy for tubal sterilization and usage of the special SEE-FIM protocol for histopathological examination of removed tubes to ensure identification of STIC was still low in 2015, but was stated by the majority of survey participants in 2022.

Analysis of the development of case numbers confirms increasing implementation of opportunistic salpingectomy at the occasion of hysterectomy into clinical routine in Germany since 2012. Consequently, the combination of hysterectomy with OS is now performed in more than two thirds of cases in women at the age of 35–49 years and has become a new standard. Salpingectomy was carried out even more frequently than appendectomy in the German female population in 2020.

Opinions of German gynecologists portrayed in our survey are in agreement with attitudes of e.g., gynecologists in Austria (Potz et al. 2016) and the United States of America (Gill and Mills 2013). However, in these countries supporting recommendations for OS have been in place for several years, while expert statements in Germany were referring to lack of long-term prospective study data (Pölcher et al. 2015; German Guideline Program in Oncology 2022). Our data reveal German gynecological surgeons as continuously aligning treatment strategies according to the snowballing scientific evidence on the prevention benefit of OS. This has made OS developing as a new de facto clinical standard accepted in clinical practice in addition to the de jure standards supported by formally developed guidelines.

There are several arguments regarding the benefit of OS in the prevention of high-grade serous tubo-ovarian cancer (HGSTOC). The scientific starting point was the identification of STICs as precursors of HGSTOC because of similar mutation pattern and as histopathological findings in removed tubes of del-BRCA mutation carriers. Association of STICs and HGSTOC was found in up to 68.4%. (Piek et al. 2001; Medeiros et al. 2006; Kindelberger et al. 2007; Carlson et al. 2008; Jarboe et al. 2009; Labidi-Galy et al. 2017). Additionally, there are mouse models which demonstrate development of STIC and HGSC following inactivation of BRCA, TP53 and PTEN. HGSTOC prevention could be achieved by early salpingectomy in these mice (Perets et al. 2013). Furthermore, tubal ligation (Cibula et al. 2011) and to a much greater extent salpingectomy has been shown to be a protective factor in population-based studies on the risk for ovarian carcinoma (Lessard-Anderson et al. 2014; Falconer et al. 2015, 2021; Madsen et al. 2015; van Lieshout, L A M et al. 2021). Subanalyses revealed that the protective effect is observed for type II (OR 0.62, 95% CI 0.45–0.85) ovarian cancer (mainly HGSTOC), but not for type I (OR 1.16, 95% CI 0.75–1.78) and is higher after bilateral (OR 0.10, 95% CI 0.01–0.71) compared to unilateral (OR 0.75, 95% CI 0.54–1.04) salpingectomy (Darelius et al. 2021). In 2022, the first population-based retrospective cohort study on the outcome after OS has confirmed the reduced risk for HGSTOC (Hanley et al. 2022).

The most important concerns regarding OS, also brought forward in this survey, are possible risks of perioperative complications and a potentially increased duration of surgery. For OS in combination with hysterectomy, this was disproved by a study at university women’s hospital Jena (Vorwergk et al. 2014).The rate of secondary tubal pathologies and reoperations was increased if tubes are preserved at the time of hysterectomy (Guldberg et al. 2013; Vorwergk et al. 2014). Some authors hypothesized that ovarian vessels within the infundibulopelvic ligaments could be damaged by cauterization due to the topographical relation of ligament and fallopian tubes. Such damage would lead to reduced ovarian function or premature menopause (< 40 years). However, if salpingectomy is performed by an experienced surgeon, there is no measurable effect on reduced ovarian reserve or function (Behnamfar and Jabbari 2017; Kotlyar et al. 2017; Asgari et al. 2018; Wang and Gu 2021; Gelderblom et al. 2022a; van Lieshout, Laura et al. 2019). Most survey participants nevertheless perform OS not before a patient age of 40 years. Two controlled prospective studies with 900 (SALSTER—SALpingectomy for STERilization) and 1200 (Gelderblom et al. 2022b) participants, respectively, are ongoing to evaluate the long-term safety of opportunistic salpingectomy regarding onset of menopause.

The progress of OS case numbers in Germany – particularly in combination with hysterectomy – reflects the confident attitude of gynecologists toward comprehensive implementation of scientific findings into clinical routine. Timing of the increase of case numbers coincides with the paradigm shift concerning the pathogenesis of HGSTOC and recognition of the role of the fallopian tubes in ovarian cancer development (Kindelberger et al. 2007; Jarboe et al. 2009; Kurman and Shih 2010; Cibula et al. 2011; Kurman et al. 2011). Similar trends have been published for the USA (Hicks-Courant 2016; Mandelbaum et al. 2020; Karia et al. 2021) and Taiwan (Ding et al. 2018). The timeframe between 2012 and 2017, when the majority of our survey participants began to implement OS at their institutions corresponds to the trend observed in the analysis from the Federal Statistical Office of Germany.

Implementation of OS in benign hysterectomy procedures may have additionally been encouraged by modern surgical approaches favoring laparoscopic assistance compared to the vaginal route. The earlier was found to improve safe accessibility of the fallopian tubes (Mothes et al. 2018). In Germany, the number of exclusively vaginal hysterectomies declined from 50% in 2005 to 21% in 2020 with the concomitant increase of laparoscopic approaches from 10 to 57% (according to Federal Statistical Office of Germany, "Statistisches Bundesamt", special analysis of DRG cases).

Prevalence of STICs in prophylactically removed fallopian tubes is undoubtedly low. In a series of 235 BRCA1/2 mutation carriers, STICs were found in two (0.9%) patients (van der Hoeven et al. 2018). German guidelines (AWMF S3-LL Version 5.1—May 2022) recommend surgical staging in case of STICs detection to exclude high-grade carcinoma (German Guideline Program in Oncology 2022). Two reviews collecting case reports identified 99 (Ruel-Laliberté et al. 2022) and 112 (Linz et al. 2022) diagnoses of isolated STIC after risk reducing or opportunistic salpingectomy. Of these patients, 83.9% (Ruel-Laliberté et al. 2022) and 86.6% respectively (Linz et al. 2022) were confirmed BRCA mutation carriers. Surgical staging was performed in 26.0% (Ruel-Laliberté et al. 2022) and 28.6% (Linz et al. 2022) of STIC cases with three (Ruel-Laliberté et al. 2022) or no (Linz et al. 2022) diagnoses of ovarian HGSC. Nine patients (9.1%) (Ruel-Laliberté et al. 2022) and eight patients (7.1%) (Linz et al. 2022), respectively, developed subsequent HGSOC after a median follow up of 58.5 (Ruel-Laliberté et al. 2022) and 42.5 (Linz et al. 2022) months, respectively. A STIC registry is currently being established in Germany to collect data on incidence, treatment and outcome.

Tubal ligation for permanent sterilization was preferably performed by most survey respondents in 2015, although the majority was convinced, that bilateral salpingectomy would be the most effective method and would have a benefit concerning cancer risk reduction. This picture has changed considerably in 2022, with 56.8% of respondents indicating bilateral salpingectomy as their preferred method of sterilization. Potentially increased intraoperative time and increased risk of intraoperative complications discouraged gynecological surgeons from performing bilateral salpingectomy. However, a recent meta-analysis demonstrated that safety and efficiency of bilateral salpingectomy are comparable to tubal ligation and should be preferred as sterilization method because of the stronger effect concerning ovarian cancer risk reduction (Mills et al. 2021). The results presented in our study focus on inpatient procedures performed in hospitals. Yet, tubal sterilization is carried out to large extent in outpatient clinics. Broad implementation of salpingectomy for permanent sterilization in Germany might currently be hindered by the increased financial expenditure for the surgery, which has to be paid by the patients out of their own pockets. Because of the reduced cancer risk, it might be cost-effective for public insurance companies to reimburse the additional costs, which arise in comparison to standard tubal ligation.

### Limitations

According to the German Hospital Society ("Deutsche Krankenhausgesellschaft", https://dkgev.deutsches-krankenhaus-verzeichnis.de), there are 759 hospitals throughout Germany performing hysterectomies. In the physicians´ statistics (“Aerztestatistik”) published by the German Medical Association (“Bundesaerztekammer”) in 2021, 733 gynecologists are working as senior physicians in hospitals. Given the approximate number of 750 possible respondents, the return rate of our survey would be 27% (2015) and 22% (2022), respectively. Consequently, a non-responder bias could distort the results of the survey. Furthermore, precise determination of physicians’ opinions could have been hampered due to the opportunity to check multiple answers and missing free text response fields. Strategy of survey dissemination was different in 2015 and 2022, resulting in limited comparability of the results. Especially the higher proportion of respondents from academic institutions and certified gynecological cancer centers could contribute to the observed higher endorsement of OS in 2022.

Reliability of case number analysis is limited by the sole consideration of in-house hospital procedures and possible inaccuracies during encoding of diagnosis related groups for billing. In addition, case number data from the Federal Statistical Office of Germany do not contain information regarding indications for surgery, whether patients were informed about the possibility of opportunistic salpingectomy and whether salpingectomy was conducted uni- or bilaterally.

## Summary and conclusion

Despite missing expert recommendations, OS is performed in Germany in combination with hysterectomies for benign indications in the majority of cases and is beginning to replace tubal ligation as procedure for permanent sterilization. This is confirmed in this study on the basis of a repeated survey of German gynecological surgeons in combination with an analysis of official German statistics between 2005 and 2020. The observed trend is not confined to academic institutions and certified cancer centers, but also occurs in primary care hospitals nationwide.

Our analysis establishes a basis for further refinement of expert recommendations and guidelines regarding opportunistic salpingectomy to shift a de facto standard to a new de jure standard. This is needed, because physicians demand a firm foundation for this procedure, which is frequently performed by now. We recommend an interdisciplinary consultation to define a consistent approach concerning counseling, indication, procedure and histopathological examination. A prospective register collecting data regarding benefit, potential risks and health economics considerations would be helpful particularly when collaborating with cancer registries. Women desiring permanent contraception should be informed in detail concerning benefit and risk of bilateral salpingectomy.

## Supplementary Information

Below is the link to the electronic supplementary material.

Supplementary file1 (DOCX 18 KB) 

Supplementary file 2 (PDF 381KB)

## Data Availability

The datasets generated during and/or analyzed during the current study are available from the corresponding author on reasonable request.
