# Supplementary material for: Ovarian cancer prevention by opportunistic salpingectomy is a new de facto standard in Germany
Source: J Cancer Res Clin Oncol. 2023 Feb 27;149(10):6953–66. doi: 10.1007/s00432-023-04578-5 (PMC10374707; doi:10.1007/s00432-023-04578-5)
Supplement: Supplementary file 1 — Supplementary file1 (DOCX 18 KB) [file 432_2023_4578_MOESM1_ESM.docx]

Salpingectomy – Supplemental tables

**Table S1:** Survey opportunistic salpingectomy - Anamnestic factors and age. Multiple answers were allowed for all questions.

|  | **2015** | | **2022** | |
| --- | --- | --- | --- | --- |
| **Questionnaire Item** | **n** | **%** | **n** | **%** |
| ***Which anamnestic factors influence your decision to indicate bilateral salpingectomy?***  Patient age  Adiposity  Comorbidities (Diabetes mellitus, metabolic syndrome)  Infertility  Endometriosis  Peri- and postmenopausal hormone replacement therapy  Possible increased risk for gynecologic cancer (family history positive for carcinosis)  Confirmed BRCA Mutation  Skipped question | 106  22  38  26  53  14  140  109  35 | 63.1  13.1  22.6  15.5  31.5  8.3  83.3  64.9 | 105  30  32  23  50  14  110  98  34 | 79.6  22.7  24.2  17.4  37.9  10.6  83.3  74.2 |
| **Starting from which age and up to which age of the patient do you perform opportunistic salpingectomy?**  From 30 years  From 40 years  From 50 years  Up to 50 years  Up to 60 years  Up to 70 years  Skipped question | 56  85  25  1  12  66  31 | 32.6  49.4  14.5  0.6  7.0  38.4 | 50  74  20  6  13  39  23 | 35.0  51.8  14.0  4.2  9.1  27.3 |

**Table S2:** Survey opportunistic salpingectomy – Histopathological Examination. Multiple answers were allowed for all questions.

|  | **2015** | | **2022** | |
| --- | --- | --- | --- | --- |
| **Questionnaire Item** | **n** | **%** | **n** | **%** |
| **Is histopathological examination of removed tubes carried out by the in-house Pathology Department or by an external institute?**  In-house Pathology Department  External institute  Skipped question | 73  126  4 | 36.7  63.3 | 84  72  10 | 53.9  46.2 |
| **Is histopathological examination of removed tubes carried out according to a special protocol (SEE-FIM protocol for detection of STIC)?**  Yes  No  Skipped question | 53  140  10 | 27.5  72.5 | 100  48  19 | 68.0  32.7 |
