# Supplementary material for: Ovarian cancer prevention by opportunistic salpingectomy is a new de facto standard in Germany
Source: J Cancer Res Clin Oncol. 2023 Feb 27;149(10):6953–66. doi: 10.1007/s00432-023-04578-5 (PMC10374707; doi:10.1007/s00432-023-04578-5)
Supplement: Supplementary file 2 — (PDF 381KB) [file 432_2023_4578_MOESM2_ESM.pdf]

Umfrage zur prophylaktischen Salpingektomie

Sehr geehrte Frau Kollegin, sehr geehrter Herr Kollege,

um eine Einschätzung zur Verbreitung der (prophylaktischen) Eileiterentfernung im Rahmen anderer Operationen in den deutschsprachigen Ländern zu erhalten, benötigen wir Ihre Mitarbeit.

Mit diesem Schreiben möchten wir Sie dafür begeistern, an einer bundesweiten Umfrage zur Salpingektomie im Rahmen gynäkologischer Eingriffe im kleinen Becken teilzunehmen. Damit Sie sich anschließend selbst ein Bild machen können, werden wir die Daten für Sie zugänglich machen. Die Umfrage erfolgt derart anonym, dass Ihr Vorgehen in Ihrer Klinik nicht mit Ihrem Namen oder dem Ihrer Klinik veröffentlicht wird.

Zur Teilnahme bitten wir Sie, den vorliegenden Fragebogen entsprechend Ihres derzeitigen klinikinternen Standards auszufüllen (Bearbeitungszeit ca. 5 Minuten). Um eine zeitnahe Auswertung der Ergebnisse zu gewährleisten, bitten wir Sie um eine baldmögliche Rückantwort, spätestens jedoch bis zum 30.09.2015. Dazu senden Sie den ausgefüllten Fragebogen per Fax: **030 - 450 564 977** an die Geschäftsstelle der NOGGO e.V. oder eingescannt per E-Mail an **studies@noggo.de** oder beantworten Sie unsere Fragen digital unter: **https://de.surveymonkey.com/s/salpingektomie**

Für Ihre Beteiligung möchten wir Ihnen im Voraus herzlich danken und freuen uns auf die gemeinsame Arbeit an diesem Projekt.

Prof. Runnebaum

Direktor UFK Jena

Prof. Sehouli

Direktor UFK Charité

Dieses Projekt ist eine Initiative der Universitätsfrauenklinik Jena in Zusammenarbeit mit der NOGGO e.V. und wird von der Kommission Ovar der AGO unterstützt.

I. Fragen zu Ihrem klinischen Hintergrund

(Mehrfachnennungen möglich, soweit sinnvoll)

1. Praktisch überwiegend tätig in:

- □ Allgemeine Gynäkologie/ Benigne Gynäkologie
- □ Gynäkologische Onkologie
- □ Urogynäkologie
- □ Reproduktionsmedizin
- □ Geburtshilfe

2. Wer bearbeitet die vorliegende Umfrage?

- □ Chefarzt, Klinikdirektor, Abteilungs- oder Sektionsleiter
- □ Oberarzt
- □ Belegarzt
- □ Facharzt
- □ Assistenzarzt mit Berufserfahrung

3. Klinische Tätigkeit in Jahren

- □ 20 und mehr
- □ 10-19
- □ 5-9
- □ < 5

4. Geschlecht

- □ Männlich
- □ Weiblich

5. Klinische Versorgungsstufe der Klinik, des Krankenhauses

- □ Krankenhaus der Maximalversorgung
- □ Krankenhaus der Schwerpunktversorgung
- □ Krankenhaus der Regelversorgung
- □ Belegklinik

6. Trägerschaft der Praxis, des Krankenhauses, der Praxisklinik

- □ Universitätsklinik
- □ Öffentliches Krankenhaus
- □ Freigemeinnütziges Krankenhaus (kirchlicher Träger)
- □ Privates Krankenhaus
- □ Private Praxisklinik

7. Zertifiziertes gynäkologisches Krebszentrum:

(ausgenommen Brustzentrum)

□ Ja □ Nein

8. Fallzahl großer gynäkoonkologischer Eingriffe:

______pro Jahr

II. Fragen zu Ihrer Meinung

9. Haben Sie jemals eine beidseitige Salpingektomie ohne Ovarektomie bei makroskopisch unauffälligen Tuben im Rahmen einer Hysterektomie wegen benigner Erkrankung durchgeführt?

□ Ja □ Nein

10. Falls ja, seit

□ mehr als 10 Jahren □ 5-10 J. □ 2-5 J. □ weniger als 2 J.

11. in der Häufigkeit

□ kleiner 10% □ 10-50% □ mehr als 50% □ immer

12. Falls ja, warum haben Sie die Hysterektomie durch eine beidseitige Salpingektomie ergänzt?

□ Um das Risiko nachfolgender chronischer Unterbauchschmerzen zu senken.

□ Um das Risiko für die Entstehung einer Karzinomerkrankung zur senken.

□ Um das Risiko für einer Karzinomerkrankung bei einer Patientin mit familiärem Brust- und Eierstockkrebsrisiko zu senken.

□ Um das Risiko für eine operative Revision zu senken.

□ Um das Risiko für die Entstehung einer Hydrosalpinx zu senken.

13. Falls nein, warum nicht?

□ Da es ein höheres Risiko für intraoperative Komplikationen birgt.

□ Da es die Operationszeit verlängert.

□ Da es das Risiko für die Entstehung einer Karzinomerkrankung nicht senken kann.

□ Da es keinen Einfluss auf das Risiko einer operativen Revision hat.

□ Da es keinen Nutzen hat.

14. Nutzen und Risiken: Bei einer Abwägung von Nutzen und Risiken der beidseitige Salpingektomie im Rahmen einer Hysterektomie ohne Ovarektomie bei benigner Indikation gehe ich davon aus, dass

□ der Nutzen die Risiken überwiegt.

□ es keinen Nutzen gibt.

15. Welche anamnestischen Faktoren beeinflussen ihre Indikationsstellung zur prophylaktischen Salpingektomie beidseits?

□ Alter der Patientin
□ Adipositas
□ Komorbiditäten (Diabetes mellitus, metabolisches Syndrom)
□ Infertilität
□ Endometriose
□ Peri- und postmenopausale Hormonersatztherapie
□ Mögliche familiäre Belastung (positive Familienanamnese für Karzinomerkrankungen)
□ Nachgewiesene BRCA Mutation

16. Ab welchem Alter und bis zu welchem Alter führen Sie eine prophylaktische Salpingektomie durch?

□ ab 30 Jahre □ bis 50 Jahre
□ ab 40 Jahre □ bis 60 Jahre
□ ab 50 Jahre □ bis 70 Jahre

17. Zusätzliche Risiken: Gehen Sie davon aus, dass zusätzliche Risiken entstehen, eine beidseitige Salpingektomie im Rahmen einer Hysterektomie oder einer Sterilisation durchzuführen?

□ Ja □ Nein

18. Der größte Nutzen: Was meinen Sie ist der größte Nutzen einer elektiven Bilateralen Salpingektomie?

□ Das Risiko für die Entstehung eines Tuben-, Ovarial- oder Peritonealkarzinoms kann gesenkt werden.
□ Das Risiko für die Entstehung chronischer Unterbauchschmerzen kann gesenkt werden.
□ Das Risiko für Tubenkomplikationen kann gesenkt werden.
□ Ich gehe davon aus, dass es keinen Nutzen gibt.

19. **Irreversible Kontrazeption: Welche Methode halten Sie für die effektivste Methode für eine irreversible Kontrazeption bei Frauen über 35 Jahren?**
    - □ Bipolare Koagulation mit oder ohne Durchtrennung der Tube bds.
    - □ Bilaterale Salpingektomie
    - □ Filshie Clip (Titan-Clip mit Silikon-Gummi-Oberfläche)
    - □ Silikon-Gummiband (Falope-Ring)
    - □ Kein signifikanter Unterschied zwischen den genannten Methoden

20. **Für welche Indikation würden Sie eine totale beidseitige Salpingektomie als Sterilisationsmethode in Erwägung ziehen?**
    - □ Bei pathologisch veränderten Tuben.
    - □ Um das Risiko für die Entstehung einer Karzinomerkrankung zur senken.
    - □ Bei familiärem Brust- und oder Eierstockskrebsrisiko, um das Risiko für die Entstehung einer Karzinomerkrankung zu senken.
    - □ Um das Risiko nachfolgender chronischer Unterbauchschmerzen zu senken.
    - □ Bei Patientinnen nach gescheiterter irreversibler Kontrazeption um ein erneutes Versagen zu verhindern.
    - □ Ich würde eine beidseitige Salpingektomie als Sterilisationsverfahren nicht durchführen.

21. **Warum würden Sie die beidseitige Salpingektomie nicht als Sterilisationsmethode in Erwägung ziehen?**
    - □ Da es die Operationszeit verlängert.
    - □ Da es ein höheres Risiko für intraoperative Komplikationen birgt.
    - □ Da es gegenüber den anderen Sterilisationsverfahren keinen Vorteil hat.
    - □ Da es keinen Nutzen hat.

22. **Welche Sterilisationsmethode bevorzugen Sie persönlich?**
    - □ Bipolare Koagulation mit oder ohne Tubendurchtrennung
    - □ Beidseitige Salpingektomie
    - □ Filshie Clip (Titan-Clip mit Silikon-Gummi-Oberfläche)
    - □ Silikon-Gummiband (Falope-Ring)

23. Sollte die beidseitige Salpingektomie einer jeden Frau empfohlen werden, die ihre Familienplanung abgeschlossen hat und eine endoskopische, abdominale oder vaginale Operation erhält, in der die Tuben erreichbar sind?

□ Ja □ Nein

24. Erfolgt die histopathologische Befundung über ein klinikinternes Institut oder extern?

□ klinikinternes Institut

□ externes Institut

25. Erfolgt die histopathologische Befundung der resezierten Tuben nach speziellen Protokollen (SEE-FIM Protokoll zur Detektion von STIC-Läsionen)**?**

□ Ja □ Nein

Vielen Dank für Ihre Teilnahme!
